# Modulation of RNase E Activity by Alternative RNA Binding Sites

**DOI:** 10.1371/journal.pone.0090610

**Published:** 2014-03-05

**Authors:** Daeyoung Kim, Saemee Song, Minho Lee, Hayoung Go, Eunkyoung Shin, Ji-Hyun Yeom, Nam-Chul Ha, Kangseok Lee, Yong-Hak Kim

**Affiliations:** 1 Department of Life Science, Chung-Ang University, Seoul, Republic of Korea; 2 Department of Genetics, Stanford University, Stanford, California, United States of America; 3 Department of Manufacturing Pharmacy, Pusan National University, Busan, Republic of Korea; 4 Department of Microbiology, Catholic University of Daegu School of Medicine, Daegu, Republic of Korea; Yonsei University, Korea, Republic Of Seoul

## Abstract

Endoribonuclease E (RNase E) affects the composition and balance of the RNA population in *Escherichia coli* via degradation and processing of RNAs. In this study, we investigated the regulatory effects of an RNA binding site between amino acid residues 25 and 36 (^24^LYDLDIESPGHEQK^37^) of RNase E. Tandem mass spectrometry analysis of the N-terminal catalytic domain of RNase E (N-Rne) that was UV crosslinked with a 5′-^32^P-end-labeled, 13-nt oligoribonucleotide (p-BR13) containing the RNase E cleavage site of RNA I revealed that two amino acid residues, Y25 and Q36, were bound to the cytosine and adenine of BR13, respectively. Based on these results, the Y25A N-Rne mutant was constructed, and was found to be hypoactive in comparison to wild-type and hyperactive Q36R mutant proteins. Mass spectrometry analysis showed that Y25A and Q36R mutations abolished the RNA binding to the uncompetitive inhibition site of RNase E. The Y25A mutation increased the RNA binding to the multimer formation interface between amino acid residues 427 and 433 (^427^LIEEEALK^433^), whereas the Q36R mutation enhanced the RNA binding to the catalytic site of the enzyme (^65^HGFLPL*K^71^). Electrophoretic mobility shift assays showed that the stable RNA-protein complex formation was positively correlated with the extent of RNA binding to the catalytic site and ribonucleolytic activity of the N-Rne proteins. These mutations exerted similar effects on the ribonucleolytic activity of the full-length RNase E *in vivo*. Our findings indicate that RNase E has two alternative RNA binding sites for modulating RNA binding to the catalytic site and the formation of a functional catalytic unit.

## Introduction

RNase E participates in the degradation and processing of RNAs in bacterial cells. Although RNase E has a preference for the cleavage of single-strand AU-rich regions within RNA substrates, the enzyme efficiency and specificity for the cleavage site seems to be affected by the stem-loop structure, membrane binding and phosphorylation status at the 5′ terminus of RNA substrates [1]–[4]. In *Escherichia coli*, RNase E (UniProt KB no., P21513) is an essential protein with a mass of 118.2 kDa and a subunit structure that consists of two distinct halves. The N-terminal domain (NTD; amino acid residues 1–529) contains the site-specific endonuclease activity that is sufficient for cell survival [5], [6]. X-ray crystallography analysis has shown that the NTD of RNase E (hereafter named N-Rne) consists of several sub-domains: an RNase H-like subdomain at the N-terminus (1–37), an RNA binding S1 domain (39–119), an Zn-binding domain (401–414) that stabilizes dimer formation, and a small domain (415–529) that mediates multimer formation at the dimer-dimer interface [7], [8]. On the other hand, the C-terminal domain (CTD; 530–1061) is composed of an unstructured scaffold domain that serves as a platform for the degradosome complex [9].

The enzymatic activity and cellular concentration of RNase E are strictly regulated through various mechanisms in *E. coli*. Adventitious overexpression or increased enzymatic activity of RNase E cause cellular toxicity leading to growth retardation [10], [11], indicating the importance of regulation of RNase E expression and activity. Although the mechanisms are incompletely understood, autoregulation is the main process that maintains RNase E cytosolic levels by cleaving the 5′ UTR of its own transcript when RNase E activity exceeds cellular needs [12]–[14]. The CTD of RNase E acts as a positive modulator to enhance endoribonuclease activity by interactions with macromolecules bound to the inner membrane [15], [16]. In the cytoplasm, the CTD of RNase E also serves as a negative modulator to reduce endoribonuclease activity by stoichiometric binding of an inhibitor protein, RraA or RraB, to distinct sites [17]–[19].

From mutagenesis studies, a hyperactive mutant with a substitution of glutamine to arginine at position 36 (Q36R) has been found to enhance RNase E activity due to a decrease in uncompetitive inhibition of the RNA substrate bound to the ^24^LYDLDIESPGHEQK^37^ peptide region [10]. This finding prompted us to investigate the regulatory effects of this region on RNase E activity. Strikingly, we found that an Y25A mutant attenuated RNase E activity both *in vivo* and *in vitro*, in contrast to the effects of the Q36R mutant. Using a radiolabeled BR13 oligoribonucleotide (p-BR13) substrate, EMSA, UV crosslinking and mass spectrometry analyses were performed to examine the molecular interactions between RNA and RNase E. This study shows that the RNase E has two alternative RNA binding sites at the N-terminal domain, which contribute to the regulation of the enzyme activity by uncompetitive and allosteric inhibition modes.

## Materials and Methods

### Strains and plasmids

The strains and plasmids used in this study are listed in Table 1. Site-directed mutagenesis was carried out by overlap extension PCR using a DNA fragment encoding the N-terminal sequence (1–398) of RNase E. The PCR products were digested with *Nar*I and *Hin*dIII (New England Biolabs Inc., Ipswich, MA), and cloned into compatible sites of pNRNE4. The primers used were Nrne1-F (5′-ACCACCCTGGCGCCCAATACGCAA-3′), Nrne1-R (5′-ATATCCAGGTCAGCCAGACGCTGCC-3′), Nrne2-F (5′-GGCAGCGTCTGGCTGACCTGGATAT-3′), and Nrne2-R (5′-TTTCAGACGGAAGCTTAAATCCCA-3′). The pLAC-RNE2-Y25A plasmid was constructed by subcloning the *Nar*I and *Hin*dIII fragment of pNRNE-Y25A into the same restriction enzyme sites in pLAC-RNE2.

**Table 1:** *E. coli* strains and plasmids used in this study.

Strains/Plasmids	Description	Reference
N3433	*lacZ43*(Fs) LAM^-^ *relA1 spoT*1 *thi*-*1*	[28]
KSL2000	Same as N3433 but *rne::cat recA::Tn10* [pBAD-RNE]	[20]
KSL2003	Same as N3433 but *rne::cat recA::Tn10* [pLAC-RNE2]	[20]
KSL2003-Q36R	Same as N3433 but *rne::cat recA::Tn10* [pLAC-RNE2-Q36R]	[10]
KSL2003-Y25A	Same as N3433 but *rne::cat recA::Tn10* [pLAC-RNE2-Y25A]	This study
pBAD-RNE	pSC101 *ori*, Km^r^, *rne* under pBAD	[20]
pACYC177	p15A *ori*, Ap^r^, Km^r^	[29]
pNRNE4	p15A *ori*, Apr, N-rne under placUV5	[22]
pNRNE4-Q36R	p15A *ori*, Apr, N-rne-Q36R under placUV5	[10]
pNRNE4-Y25A	p15A *ori*, Apr, N-rne-Y25A under placUV5	This study
pLAC-RNE2	pSC101 *ori*, Ap^r^, *rne* under placUV5	[20]
pLAC-RNE2-Q36R	pSC101 *ori*, Ap^r^, *rne-*Q36R under placUV5	[10]
pLAC-RNE2-Y25A	pSC101 *ori*, Ap^r^, *rne-*Y25A under placUV5	This study
pET28a	pMB1 *ori*, Km^r^	Novagen

### *In vitro* cleavage of RNase E substrate

BR13 was 5′-end labeled with [γ-^32^P]-ATP using T4 polynucleotide kinase (Takara, Japan) and the labeled products (p-BR13) were purified by MicroSpin™ G25 columns (GE Healthcare, UK) according to the manufacturer's instructions [20]. Approximately 2 pmol of p-BR13 was incubated with 1 pmol of purified wild-type N-Rne or Y25A or Q36R mutant proteins at 37°C in 20 µl of 200 mM Tris-HCl buffer (pH 8.0) containing 1 M NaCl, 1 mM DTT, 50 mM MgCl_2_, and 50% (v/v) glycerol. The reaction products were separated in 15% denaturing polyacrylamide gels.

### Electrophoretic mobility shift assay (EMSA)

Approximately 0.5 pmol of p-BR13 was incubated with increasing protein concentrations of wild-type N-Rne or the Y25A and Q36R mutants for 10 min at 4°C or room temperature in 20 µl of 10 mM Tris-HCl buffer (pH 8.0) containing 0.1 mM DTT, 1.0 mM EDTA, and 10% (v/v) glycerol. The reaction products were separated using TBE native gels composed of 8% acrylamide/bisacrylamide solution (19∶1) and 2.5% glycerol in 1× Tris-Borate-EDTA buffer.

### UV-crosslinking assay

Twenty pmol of wild-type N-Rne, Y25A, or Q36R mutant protein was mixed with 20 pmol of p-BR13 in 20 µl of 10 mM Tris-HCl buffer (pH 7.5) containing 0.1 mM DTT, 1.0 mM EDTA and 10% (v/v) glycerol, and then exposed to UV light (254 nm) using a CL1000 Ultraviolet Cross Linker (UVP) for 30 min at room temperature. The RNA-protein complexes induced by UV-crosslinking were examined in autoradiograms of 10% SDS-PAGE gels including lanes for experimental controls that were prepared under the same conditions without the addition of p-BR13 or without UV irradiation.

### Liquid chromatography-tandem mass spectrometry analysis

After UV crosslinking, proteins were stained with Coomassie Blue and monomer bands were excised from the gel to avoid complex errors from UV crosslinking between proteins or RNAs. The destained gel slices were treated twice with 50 mM NaOH at 60°C for 15 min with an Eppendorf Thermomixer in order to remove the phosphodiester bond of ribonucleotides as described previously [10]. The washed gels were reduced with 10 mM DTT at 60°C for 10 min, and then alkylated with 55 mM iodoacetamide at room temperature in the dark, and subsequently washed with 100 mM ammonium bicarbonate (AmBic), 50% acetonitrile (ACN)-AmBic, and 100% ACN. The dried gels were subjected to enzyme digestion with a sequencing-grade trypsin (Promega) for 24 h at 37°C, followed by overnight digestion with chymotrypsin (Roche) according to the manufacturer's protocols. Peptides were extracted, dried *in vacuo*, and dissolved in 0.4% acetic acid for LC-tandem mass spectrometry analysis as described previously [10]. To identify and quantitatively analyze peptides that were crosslinked with p-BR13, the samples were analyzed using a sensitive LTQ Velos mass spectrometer (Thermo Fisher Scientific Inc.) equipped with an EASY-nLC 1000 system and a reverse-phased Magic C18AQ capillary column (75 µm×75 mm). The LC condition used was a 90-min linear gradient from 5% to 40% ACN in a 0.1% formic acid buffer solution, followed by a 10 min column wash with 80% ACN and a 20 min re-equilibration to the initial buffer condition. A full-scan survey was carried out between *m/z* 300–2,000 and was followed by nine data-dependent scans of the most intense ions with the following options: isolation width, ±1.5 *m/z*; collision energy, 35%; and dynamic exclusion duration, 30 sec. The resultant mass data were analyzed using Proteome Discoverer version 1.3 with a combined database of N-Rne and its mutant peptide sequences, *E. coli* K-12 proteins (Swiss-Prot) and the common Repository of Adventitious Proteins (downloaded from URL ftp://ftp.thegpm.org/fasta/cRAP) that are present either by accident or through unavoidable contamination of the protein samples. Tandem mass spectra were analyzed using the Sequest algorithm [21] with the following options: 1 miscleaved site from digestion with trypsin and chymotrypsin; precursor mass tolerance, 200 ppm; fragment mass error, 1 Da; variable modifications for carbamidomethylation (cysteine), oxidation (cysteine, methionine, or tryptophan), and UV crosslinking of any amino acids with the bases of ribonucleotides, adenine (+267.1 Da), guanine (+283.1 Da), cytosine (+243.1 Da) or uracil (+244.1 Da); and filtering with FDR<0.05, Xcorr >1.5, and SpScore >200. Extracted ion chromatograms (XICs) of the identified peptides were analyzed using the QualBrowser program version 2.0.7 (Thermo Fisher Scientific Inc.) with a precursor ion mass (*m/z*) tolerance of 200 ppm. All samples were analyzed at least in duplicate.

### Circular Dichroism

Purified N-Rne proteins were dialyzed in storage buffer (20 mM Tris-HCl, pH 7.5, 100 mM NaCl, 0.1 mM EDTA, 60% glycerol). Spectra were collected in the range of 340–200 nm at intervals of 1 nm, with three accumulations being recorded on a JASCO J-715 spectropolarimeter.

## Results

### Effects of N-Rne mutants on the growth of *E. coli*

**Figure 1:**
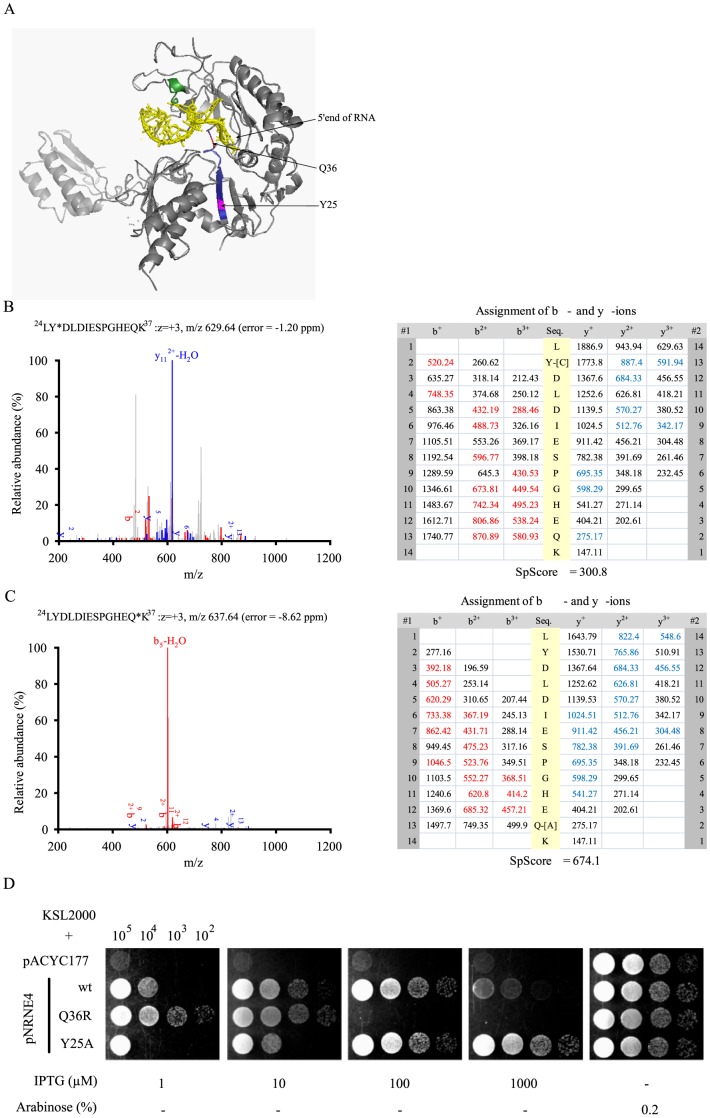
Identification of a hypoactive N-Rne mutant. (A) Location of the isolated single amino-acid substitutions in the crystal structure of the N-terminal region of RNase E. Two tryptic peptides that were UV-crosslinked to p-BR13, ^24^LYDLDIESPGHEQK^37^ and ^65^HGFLPLK^71^, are colored in blue and green, respectively. p-BR13 is colored in yellow. The diagram was generated using PyMOL software. (B) Tandem mass spectrum assigned to the predicted *b*- and *y*-ions generated by collision-induced fragmentation of the peptide, ^24^L**Y^C^**DLDIESPGHEQK^37^, with the Y25 residue bound to cytosine (*m/z* = 629.63, *z* = +3, mass error  = −1.20 ppm). (C) Tandem mass spectrum assigned to the predicted *b*- and *y*-ions generated from collision-induced fragmentation of the peptide, ^24^LYDLDIESPGHE**Q^A^**K^37^, with the Q36 residue bound to adenine (*m/z* = 637.64, *z* = +3, mass error  = 8.62 ppm). (D) Growth characteristics of cells expressing wild-type N-Rne or the Q36R or Y25A mutant proteins. Growth of KSL2000 cells harboring pNRNE4, pNRNE4-Q36R, or pNRNE4-Y25A was measured individually on LB-agar plates containing 1.0 to 1000 µM IPTG. KSL2000 harboring pACYC177 grew only when full-length RNase E was expressed from pBAD-RNE in the presence of 0.2% arabinose. Numbers on the top indicate the number of bacterial cells in each spot.

In a previous study, UV crosslinking and mass spectrometry analysis showed that p-BR13 binds to the peptide, ^24^LYDLDIESPGHEQK^37^, leading to the uncompetitive inhibition of N-Rne activity [10]. This peptide region is located in an RNase H fold unit (protomer B) of a 5′ sensor pocket and is contacted on one side by an RNA binding S1 domain of N-Rne [7], [8]. In this study, we generated tandem mass spectral data supporting the alternative RNA binding to specific amino acid residues in the structure model of N-Rne (Figure 1A). Collision-induced dissociation electrospray ionization tandem mass spectra of the p-BR13-bound N-Rne showed the predicted fragment ions of the peptide with the respective Y25 and Q36 residues bound to cytosine and adenine of p-BR13, respectively (Figure 1B–C). These spectra were not generated from the N-Rne protein containing the Q36R mutation (data not shown).

To further examine the functional role of the alternative RNA binding site in N-Rne, the Y25 residue was substituted with an alanine codon in the pNRNE4 plasmid, and the resulting plasmid was used to transform *E. coli* strain KSL2000. The Y25 residue was chosen for its potential to make direct contact with p-BR13 as it is in close proximity to the only cytosine of p-BR13. This strain contains a deletion of the chromosomal *rne* gene, which is complemented by expression of full-length RNase E from a cloned copy of *rne* under the control of an arabinose-inducible promoter (pBAD-RNE) [20], [22]. When RNase E production was induced by 0.2% arabinose, the KSL2000 cells showed similar growth, regardless of the presence of the wild-type or mutant pNRNE4 plasmid (Figure 1D). Without arabinose, however, the growth of KSL2000 strains containing a wild-type or mutant pNRNE4 plasmid varied depending on expression levels of N-Rne proteins, which were controlled by the addition of different concentrations of IPTG (1.0 to 1,000 µM) to the culture medium. Compared with the wild-type N-Rne, the Y25A mutant (N-Rne-Y25A) reduced cell viability and growth by one or two orders of magnitude below the wild-type N-Rne induced by up to 100 µM IPTG, which is equivalent to the 0.2% arabinose that induced full-length RNase E from the pBAD-RNE plasmid. A 1,000 µM concentration of IPTG had adverse effects on the growth of cells with wild-type N-Rne, but not that of cells with the Y25A mutant protein. This indicates that the Y25A mutant has a negative effect on N-Rne activity. As previously shown [10], the Q36R mutant exhibited hyperactivity that enabled and supported cell viability and growth at 100- to 1000-fold lower doses of IPTG than the wild-type N-Rne and N-Rne-Y25A. The phenotype of N-Rne-Y25A was unexpected since reduction of RNA binding to the previously proposed uncompetitive inhibition site (^24^LYDLDIESPGHEQK^37^) enhanced RNase E activity.

### *In vivo* and *in vitro* RNA cleavage activity of wild-type and mutant N-Rne proteins

**Figure 2:**
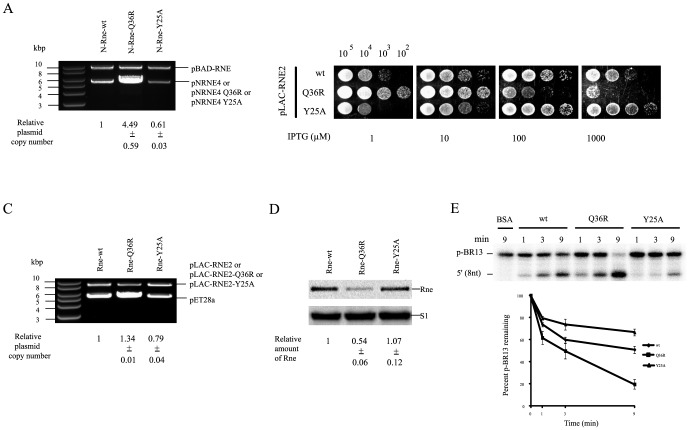
Effects of Y25A and Q36R on the catalytic activity of RNase E *in vivo* and *in vitro*. (A) Plasmid copy number of pNRNE4, pNRNE4-Q36R and pNRNE4-Y25A in KSL2000. Plasmids were purified from KSL2000 cells harboring pNRNE4, pNRNE4-Q36R or pNRNE4-Y25A and were digested with *Hin*dIII, which has a unique cleavage site in all of the plasmids tested. Plasmid copy number was calculated relative to the concurrent presence of the pSC101 derivative (pBAD-RNE), which replicates independently of Rne, by measuring the molar ratio of the ColE1-type plasmid to the pBAD-RNE plasmid. (B) Growth characteristics of KSL2003 cells expressing wild-type N-Rne or the Q36R or Y25A mutant proteins. Growth of KSL2003 cells harboring pLAC-RNE2, pLAC-RNE2-Q36R, or pLAC-RNE2-Y25A was measured individually on LB-agar plates containing 1.0 to 1000 µM IPTG. Numbers on the top indicate the number of bacterial cells in each spot. (C) Plasmid copy number of pET28a in KSL2003. Plasmids were purified from KSL2003, KSL2003-Q36R or KSL2003-Y25A cells harboring pET28a and digested with *Hin*dIII, which has a unique cleavage site in all the plasmids tested. Plasmid copy number was calculated relative to the concurrent presence of the pSC101 derivative (pLAC-RNE2, pLAC-RNE2-Q36R or pLAC-RNE2-Y25A) by measuring the molar ratio of the ColE1-type plasmid to the pSC101-derived plasmid. (D) Expression profiles of Rne and mutant proteins in KSL2003. The membrane probed with an anti-Rne polyclonal antibody was stripped and reprobed with an anti-S1 polyclonal antibody to provide an internal standard. The relative abundance of protein was quantified by setting the amount of wild-type Rne to 1. KSL2003 cells were grown in LB medium containing 10 µM IPTG. (E) *In vitro* cleavage of p-BR13 by wild-type N-Rne, Q36R and Y25A mutant proteins. Two pmol of 5′ end-labeled p-BR13 was incubated with 1 pmol of purified wild-type N-Rne or Q36R or Y25A mutant protein in 20 µl of cleavage buffer at 37°C. Samples were removed at each indicated time point and mixed with an equal volume of loading buffer. Samples were denatured at 65°C for 5 min and loaded onto 15% polyacrylamide gel containing 8 M urea. The radioactivity in each band was quantified using a phosphorimager and OptiQuant software.

RNase E is able to cleave RNA I, which acts as an antisense repressor of ColEI-type plasmid replication [23]. *E. coli* KSL2000 cells carrying both a ColE1 origin plasmid (pNRNE4) and a pSC101 origin plasmid (pBAD-RNE) have been used to assess the *in vivo* activity of RNase E against RNA I by measuring the relative copy number of pNRNE4 to pBAD-RNE when the N-Rne encoded on pNRNE4 is conditionally expressed by IPTG in the absence of arabinose [23]-[25]. In this work, when Y25A mutant protein production was induced by 100 µM IPTG, the mutant pNRNE4 plasmid copy number was 1.5-fold lower than that of the wild-type N-Rne plasmid levels, while the Q36R mutant plasmid resulted in an approximately 4.5-fold higher copy number than that of the wild-type N-Rne plasmid (Figure 2A). These results indicate that the Y25A and Q36R mutant proteins have opposite effects on RNA I cleavage.

To investigate whether the hypoactive phenotype of the Y25A mutant was maintained in the full-length Rne protein, Y25A mutation was introduced into the pLAC-RNE2 plasmid, which expresses a full-length RNase E (Rne) under control of the IPTG-inducible *lacUV5* promoter. The resulting plasmid was used to transform *E. coli* strain KSL2000. Introduction of an incompatible ampicillin resistance (Ap^r^) plasmid expressing RNase E-Y25A with a hexahistidine tag at the C-terminus under the control of the IPTG-inducible *lac*UV5 promoter (pLAC-RNE2-Y25A) into KSL2000, and selection for the incoming plasmid by growing transformants containing both plasmids (pBAD-RNE and pLAC-RNE2-Y25A) in the presence of ampicillin (50 µg/ml) and 100 µM IPTG for 40 generations, resulted in displacement of the resident Km^r^ plasmid by the Ap^r^ RNe-Y25A-expressing construct, as indicated by both the antibiotic resistance phenotype and restriction enzyme analysis of plasmid DNA. The resulting KSL2003-Y25A strain was tested for viability and growth on LB-agar containing different concentrations of IPTG, which controls RNase E-Y25A expression. The addition of 1,000 µM IPTG had adverse effects on the viability and growth of cells with full-length wild-type Rne, but not on cells with the Y25A mutant protein (Figure 2B). This indicates that a negative effect of the Y25A mutant on Rne activity is not specific to the truncated form of RNase E. To test the ability of the mutant RNase E protein to cleave RNA I, a ColE1-type test plasmid (pET28a) was introduced into the KSL2003 strain and its derivatives, and the relative plasmid copy number of pET28a to the pLAC-RNE2-derived plasmid was measured. Cells producing Rne-Y25A showed a 1.5-fold lower pET28a copy number relative to that observed in cells expressing wild-type Rne, while the pET28a copy number in the cells expressing Rne-Q36R resulted in an approximately 1.6-fold higher copy number than that of pET28a in cells expressing wild-type Rne (Figure 2C). These data indicate that the Y25A and Q36R mutants have opposite effects on RNA I cleavage when maintained in the full-length RNase E *in vivo*. Western blot analysis of Rne proteins showed that the abundance of the full-length Rne-Y25A is similar to the wild-type level, while the Rne-Q36R expression was decreased by approximately two-fold as previously reported (Figure 2D) [10].

In order to assay the enzymatic activity of wild-type and mutant N-Rne proteins *in vitro*, 5′-^32^P-end-labeled BR13 (p-BR13) was utilized as the substrate, and the cleavage products were resolved on PAGE gels and analyzed by autoradiography (Figure 2E). When the remaining substrate and 5′ product (8 nt) formed were observed during a 9-min incubation with the same concentrations of purified proteins, the Y25A mutant resulted in approximately 1.3-fold lower activity than the wild-type N-Rne, whereas the Q36R mutant displayed a 2.6-fold higher activity than the wild-type N-Rne. This implies that the Y25A and Q36R mutant proteins resulted in hypoactivity and hyperactivity, respectively, compared to wild-type N-Rne. Given their opposite effects on RNA I cleavage for the repression of pNRNE4 replication, the Y25A and Q36R mutations could influence the decay and processing of RNA substrates to a greater extent in the cells, as demonstrated by the hypo- and hypersensitivity to cell viability and growth.

### RNA binding and UV crosslinking of wild-type and mutant N-Rne proteins

**Figure 3:**
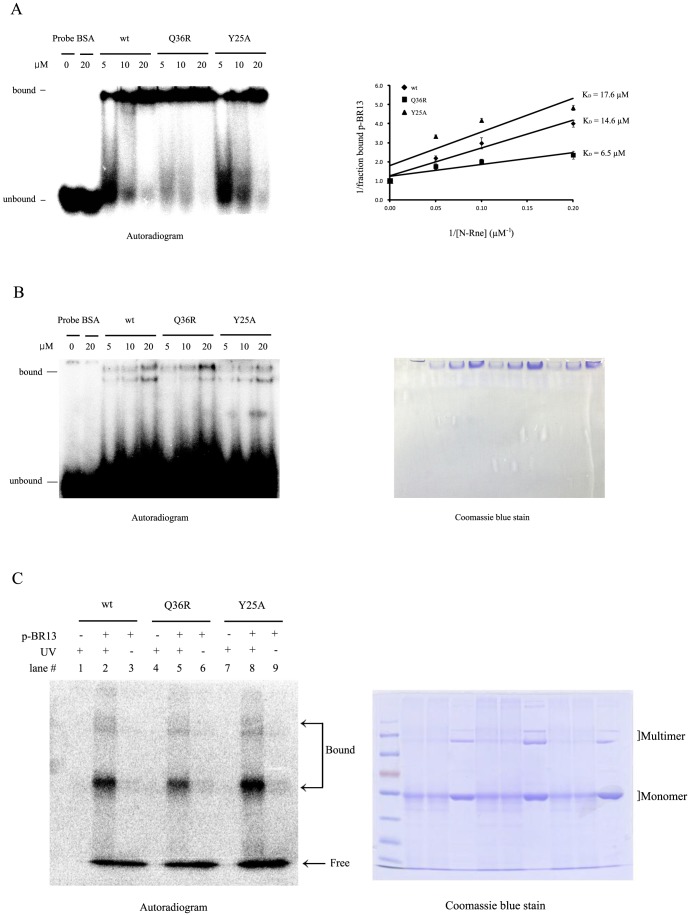
Effects of mutant proteins on RNA binding activity. (A, B) Electrophoretic mobility shift assay. The 5′ end labeled p-BR13 (0.5 pmol) was incubated with increasing concentrations of purified wild-type N-Rne or Q36R or Y25A mutant protein in 20 µl of EMSA buffer, incubated on ice (A) or at room temperature (B) for 10 min, and analyzed by 12% nondenaturing PAGE. Binding constants were calculated based on slopes calculated from the graph. To avoid induction of RNA cleavage, Mg^2+^ was omitted from the EMSA reactions. (C) UV crosslinking of N-Rne-wt, N-Rne-Q36R and N-Rne-Y25A to p-BR13. Two pmol of p-BR13 was incubated with 100 pmol of N-wild-type Rne, Q36R or Y25A mutant protein in 20 µl of crosslinking buffer and exposed to UV light for 30 min. Samples were loaded onto 10% polyacrylamide gels (lanes 2, 5, 8) and samples in the absence of p-BR13 (lane 1, 4, 7) or UV irradiation (lanes 3, 6, 9) were also loaded as controls. The gel was stained with Coomassie brilliant blue and dried. The radioactivity in each band was detected using a phosphorimager and OptiQuant software. The number of crosslinked p-BR13 per pmol of protein was calculated.

To explore the potential change in RNA substrate binding to the wild-type N-Rne and mutant active site channels, electrophoretic mobility shift assays (EMSA) were performed by mixing various concentrations of the purified proteins with a fixed concentration of p-BR13. To estimate the protein-RNA complex strength, the dissociation constant (*K*_D_) was determined by measurements of the free to bound RNA concentration ratio, as described previously [10]. The *K*_D_ value of the Y25A mutant (17.6 µM) was determined to be 1.2-fold higher than that of the wild-type N-Rne, (14.6 µM), and the Q36R mutant exhibited a low *K*_D_ (6.5 µM) compared to that of the wild-type N-Rne and the Y25A mutant (Figure 3A). The protein-RNA complex dissociation constants were inversely proportional to the association constants for the protein binding affinities to the RNA substrate, relating to the *in vivo* and *in vitro* enzyme activities. Hence, the Y25A and Q36R mutant proteins might exert opposite effects on the binding of the RNA substrate to the catalytic site of N-Rne by modification or modulation of the protein structure. We also observed that faster-migrating bands between bound- and unbound-p-BR13 bands when the reaction mixtures were incubated at room temperature before electrophoresis (Figure 3B). These bands appear to represent p-BR13 molecules that were initially bound to N-Rne proteins and dissociated during electrophoresis. The amounts of these dissociated p-BR13 molecules from wild-type and Y25A mutant proteins were approximately 2.5-fold higher than that from N-Rne-Q36R. The Q36R mutant showed enhanced catalytic activity by reducing RNA binding to the uncompetitive inhibition site of the ^24^LYDLDIESPGHEQK^37^ peptide, as shown by tandem mass spectrometry. However, it was unclear how the Y25A mutation exerted an adverse effect on N-Rne with an increased dissociation constant of the enzyme-RNA complex.

In order to examine the specific or non-specific binding of the N-Rne proteins to p-BR13, which positively or negatively affected the enzyme activity, UV crosslinking was performed to generate a covalent bond between an amino acid residue and a base of the RNA that are in close contact [26]. UV crosslinking is a useful technique since the crosslink is likely determined by the geometry and photoreactivity of the nucleotide and the corresponding amino acid. In addition, conformational changes of single-strand RNAs and the protein allow for crosslinking within the photoexcitation timescale [27]. When purified wild-type N-Rne and the Y25A and Q36R mutants (20 pmol each) were exposed to high-energy UV (254 nm) for 30 min at room temperature, they produced some non-specific bands possibly due to UV-induced crosslinking between RNAs or between proteins in the presence or absence of p-BR13 (Figure 3C). To eliminate non-specific bands, the efficiency of UV-crosslinking between protein and p-BR13 was determined from monomer bands on an SDS-PAGE gel using autoradiography. The autoradiogram showed that the Y25A mutant protein increased the molecular ratio of p-BR13-bound protein (0.016 pmol per 1 pmol unit of protein) by 1.4-fold compared to that (0.012) of the wild-type N-Rne (Figure 3C, lane 2 vs. lane 8). In contrast, the Q36R mutant resulted in a 1.4-fold decrease in the level of the p-BR13-bound protein compared to that of the wild-type N-Rne (Figure 3C, lane 2 vs. lane 5). These results contradict those determined from EMSA, where more RNA crosslinking to the protein showed a higher dissociation constant for the enzyme-RNA substrate complex.

### Mass spectrometry analysis of RNA binding sites in the wild-type N-Rne and mutant proteins

**Figure 4:**
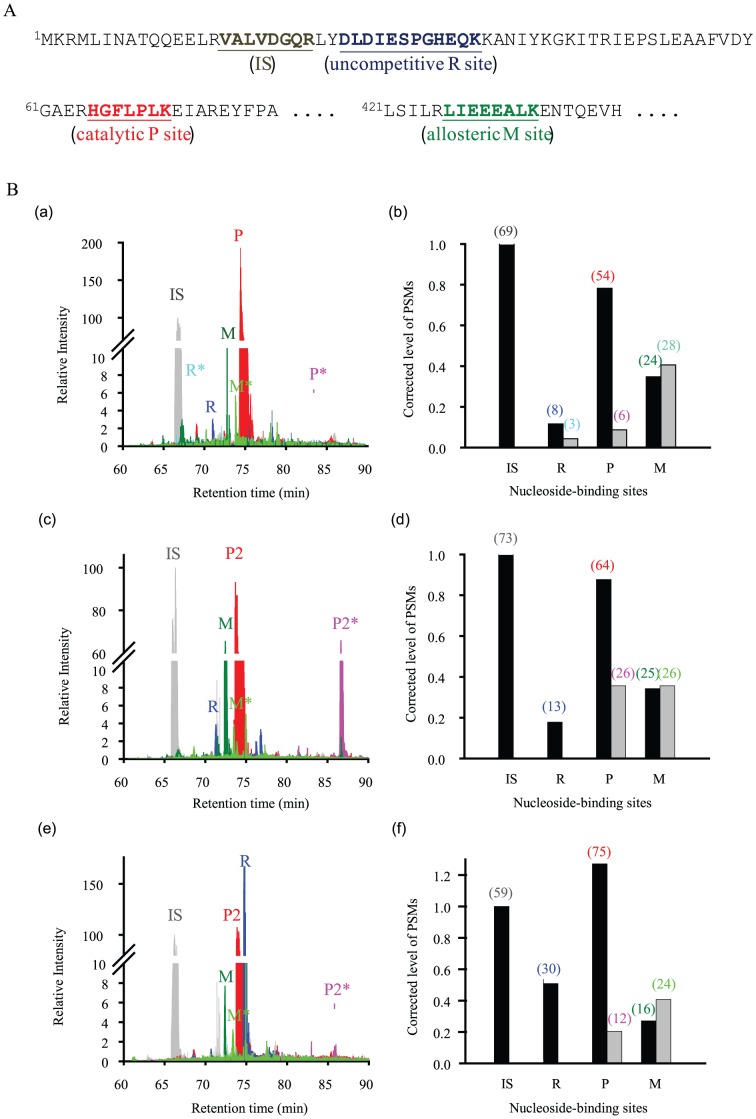
Mass spectrometry analysis of wild-type and mutant N-Rne proteins obtained from UV-crosslinking. (A) Partial peptide sequence of the wild-type N-Rne showing the regions of an internal standard (IS) sequence and nucleoside-bound peptides, denoted as R, P, and M sites, corresponding to an uncompetitive site (a.a. 26–37), a catalytic site (a.a. 65–71) and an allosteric site (a.a. 427–433), respectively. (B) Extracted ion chromatograms (XICs: panels a, c and e) and corrected PSM levels (panels b, d and f) of nucleoside-bound peptides and their parent peptides of wild-type N-Rne (a and b) and Q36R (c and d) and Y25A (e and f) mutants. Nucleoside-bound peptide peaks in the XICs are denoted with asterisks to the right of the symbols, R, P, and M, corresponding to the trypsin/chymotrypsin-digested peptides, ^26^DLDIESPGHEQK^37^, ^65^HGFLPLK^71^, and ^427^LIEEEALK^433^, respectively, in the sequence of the wild-type N-Rne. The Y25A and Q36R mutants replace the R sequence with ^24^LADLDIESPGHEQK^37^ and ^26^DLDIESPGHER^36^, respectively. In the right panels, the numbers of peptide spectrum matches (PSMs) of the parental and nucleoside-bound peptides are shown in parentheses above the black and gray bars of the corrected PSM levels. The relative levels of internal standard (IS), ^16^VALVDGQR^23^, are expressed as 100 and one unit for the calculation of relative intensity of XIC and correction of PSM levels, respectively. Tandem mass spectrometry data are given in Table S1 and Figure S2 in File S1.

The results of UV crosslinking of RNA to N-Rne proteins may stem from transient RNA binding, which can be detected by UV crosslinking and not by EMSA. This notion implies additional RNA binding sites in N-Rne. To test this hypothesis, monomer bands of wild-type, Y25A and Q36R mutant N-Rne proteins were excised from replicate gels and analyzed using an nLC-tandem mass spectrometer. The mass spectrometry data were highly reproducible in a broad dynamic range (Figure S1 in File S1). From tandem mass spectral data, each sample resulted in a total of 1,129 to 1,646 peptide spectrum matches (PSMs) of 44 to 49 unique peptides, which covered 65.94 to 71.49% of the protein sequences (Table S1 in File S1). Among these peptides, the three peptides with the asterisked residues, ^26^D*L*DIESPGHEQK^37^, ^65^HGFLPL*K*^71^, and ^427^LIEEEALK*^433^, were identified as being bound to nucleotides. The tandem mass spectra of the parental and nucleoside-bound peptides that were assigned to the predicted *b*- and *y*-ions generated from collision-induced fragmentation are shown in Figure S2 in File S1. Even though it is hard to identify which bases are crosslinked, except for the sole cytosine at the fifth position of BR13, the spectra show that the RNA bound not only to the catalytic (P) site, but also to alternative sites, an uncompetitive inhibition (R) site of the RNase H fold unit (protomer B) and a conformational (M) site at the multimer formation interface, as shown in Figure 4A. These sites were considered to be useful for the investigation of different Y25A and Q36R mutant effects on RNA binding to N-Rne.

To compare RNA binding potentials, extracted ion chromatograms (XICs) of the parental and nucleoside-bound peptides were generated by normalizing the signal intensity with the peak of a unique stripped peptide, ^16^VALVDGQR^23^, as the internal standard (**IS**) (Figure 4, panels a, c, and e). The relative levels of the IS to the total PSMs were useful for the analysis of technical errors as well as for the correction of any errors present in any sample. In this work, the relative IS values of the three samples ranged from 0.044 for the Y25A mutant to 0.061 for the wild-type N-Rne, indicating that the normalized errors did not exceed 39% in any of the samples. The error correction was simply made by dividing the PSMs of a unique peptide by the PSMs of the IS, as shown in panels b, d and f of Figure 4. The corrected PSM levels and XIC peaks showed that the Y25A and Q36R mutants changed the relative levels of the RNA-bound and -unbound peptides. An RNA-bound R peptide, ^26^D*L*DIESPGHEQK^37^, with D26 and L27 residues crosslinked with adenine and cytosine, respectively, was consistently found in wild-type N-Rne, but was not seen in the Y25A and Q36R mutant proteins. In contrast, an enhanced level of the RNA-bound P peptide, ^65^HGFLPL*K*^71^, with the L70 and K71 residues crosslinked with uracil and adenine, respectively, resulted only from the Q36R mutant protein. The changes caused by the Q36R mutation in N-Rne provided evidence for the previous suggestion that the Q36R mutation enhances endonuclease activity of N-Rne by the elimination of an uncompetitive inhibition of RNA at the R site [26]. The Y25A mutation was also able to abolish the RNA binding to the R site, but it increased the RNA binding level of a conformational M site, ^427^LIEEEALK*^433^, with the K433 residue crosslinked with cytosine. Although the actual mechanism is unclear, an RNA-bound M site at the multimer formation interface appeared to cause an allosteric inhibition by lowering the substrate binding affinity of the enzyme, as demonstrated by EMSA, UV crosslinking and *in vivo* and *in vitro* enzyme assays. We compared the structure of N-Rne-Y25A and N-Rne-Q36R proteins with wild-type N-Rne using circular dichroism (CD) to determine whether the mutations that alter the binding and enzymatic activity of the mutant N-Rne proteins lead to the misfolding of the protein. As shown in Figure S3 in File S1, the CD spectra of mutant N-Rne proteins were nearly identical to that of wild-type N-Rne, indicating that there is no significant collapse or misfolding of the mutant protein.

## Discussion

Tandem mass spectrometry analysis of N-Rne fragments that were UV crosslinked to p-BR13 has shown that the ^24^LYDLDIESPGHEQK^37^ peptide includes an alternative RNA binding site of RNase E at the Y25 and Q36 residues bonded with cytosine and adenine, respectively (Figures 1B–C). This study confirmed the previous mutagenesis study that the Q36R mutation in N-Rne enhances the catalytic activity of RNase E, but reduces the total RNA binding level by the reduction of an uncompetitive inhibition of RNA [10]. In contrast, we found that the Y25A mutation induces an adverse effect on N-Rne, because it reduces the catalytic activity of RNase E with an increase in the total RNA binding level at the conformational site of the dimer-dimer interface. Our study demonstrates that the N-terminal domain of RNase E has two alternative RNA binding sites involved in the regulation of the enzyme activity by uncompetitive and allosteric inhibition modes.

The N-Rne structure consists of several subdomains not only for the catalytic activity, but also for the regulation of a conformational change of the catalytic unit [7]–[9]. An uncompetitive RNA binding site is present in an RNase H-like subdomain at the N-terminus [10]. The previous X-ray crystallography studies showed that this site is located between the S1 subdomain and the 5′ sensing pocket region that appears to be critical for RNA binding and cleavage orientation [7], [8], and has been proposed to be an uncompetitive RNA binding site [10]. In the present study, we confirmed the binding of RNA to the proposed uncompetitive site at the N-terminus by using UV crosslinking and nLC-tandem mass spectrometry. Under a UV light, crosslinks between RNA and protein can occur at locations where there is close proximity. Even though the protein-RNA crosslinks are artificial, irreversible products that could quench the molecules and thus perturb the dynamic equilibrium of the molecular interaction, the frequency of crosslinking is correlated with the distance and the ability of the molecules to undergo a transient conformational change to a conformation that would allow crosslinking within the timescale of photoexcitation [26]. Thus, this technique is useful for identifying a target molecule or covalent bond of a photoreactive substrate like RNA by tandem mass spectrometry [21], [27]. However, there is the drawback that crosslinking can occur non-specifically between RNA bases and between proteins during photoexcitation or even due to loose binding but proximal location. Thus, we performed electrophoretic mobility shift assays (EMSAs) to determine the equilibrium dissociation constant of the enzyme-substrate (ES) complex. The faster-migrating bands between RNA-bound and -unbound proteins appear to account for the discrepancy observed with the UV crosslinking, due to loose binding at an allosteric site of the Y25A mutant. The Y25A mutation in the N-terminal domain of RNase E may induce a conformation change of the enzyme, presumably enabling RNA binding to an allosteric site of the dimer-dimer interface. This site-directed mutagenesis reveals a novel allosteric site that binds to RNA, by which RNase E is modulated in order to reduce the substrate-binding affinity.

In conclusion, our findings suggest that RNase E involves two alternative RNA binding sites in the regulation of the N-terminal catalytic domain by an uncompetitive or allosteric inhibition. The two mutants, Y25A and Q36R, abolished the RNA binding to an uncompetitive site of N-Rne, but these mutations demonstrated reciprocal effects of hypoactivity and hyperactivity, respectively, in comparison with the wild-type N-Rne and the full-length RNase E *in vivo* and *in vitro*. The Y25A mutation induces a conformational change to increase the RNA binding to an allosteric site for the inhibition of the enzyme, whilst the Q36R mutation reduces the RNA binding to an uncompetitive site, thereby resulting in increased enzyme activity. Thus, these mutations appear to be mutually independent. Taken together, the two alternative RNA binding sites of RNase E can have positive and negative effects on the stabilization and interaction of RNase E with an RNA substrate, which modulates RNase E by uncompetitive or allosteric inhibition.

## Supporting Information

File S1 **Supporting Figures S1–S3 and Table S1. Figure S1. Base peak chromatograms of UV-crosslinked monomer samples of wild-type and mutant N-Rne proteins.** Analyzed by nLC-LTQ Velos mass spectrometry between 60 to 90 min over a mass range of 300 to 2,000. After UV-crosslinking, the monomer bands were excised from SDS PAGE gels as described in the main text. **Figure S2. Tandem mass spectra and assignment of** ***b*** **- and** ***y*** **-ions of peptides bound to p-BR13.** The parent peptides, ^26^DLDIESPGHEQK^37^, ^65^HGFLPLK^71^, and ^427^LIEEEALK^433^, are denoted as R, P, and M, respectively, and show an asterisk to the right of the letter to indicate the chemical binding of nucleosides by UV-crosslinking between protein and p-BR13 in the extracted ion chromatograms (panels a, c and e) and the corrected PSM levels (panels b, d and f). A unique stripped peptide,^16^VALVDGQR^23^, used as the internal standard (**IS**) for calculation of relative intensity or corrected PSM level was included as below. (A) Tandem mass spectrum assigned to the predicted *b*- and *y*-ions generated from collision-induced fragmentation of the stripped IS peptide, ^16^VALVDGQR^23^, at *m/z* = 429.5 in a charge state of +2. (B) Tandem mass spectrum assigned to the predicted *b*- and *y*-ions generated from collision-induced fragmentation of a parental R_WT_ peptide of the wild-type N-Rne, ^26^DLDIESPGHEQK^37^, detected at *m/z* = 684.7 in the charge state of +2. (C) Tandem mass spectrum assigned to the predicted *b*- and *y*-ions generated from collision-induced fragmentation of the R_WT_* peptide, ^26^ **D** ^A^ **L** ^C^DIESPGHEQK^37^, detected at *m/z* = 627.0 in the charge state of +3 with the D26 and L27 residues bound to adenine (A) and cytosine (C), respectively. (D) Tandem mass spectrum assigned to the predicted *b*- and *y*-ions generated from collision-induced fragmentation of a parental R_Y25A_ peptide of the Y25A mutant, ^24^LADLDIESPGHEQK^37^, detected at *m/z* = 776.6 in the charge state of +2. (E) Tandem mass spectrum assigned to the predicted *b*- and *y*-ions generated from collision-induced fragmentation of a parental R_Q36R_ peptide of the Q36R mutant, ^26^DLDIESPGHER^36^, detected at *m/z* = 634.5 in the charge state of +2. (F) Tandem mass spectrum assigned to the predicted *b*- and *y*-ions generated from collision-induced fragmentation of a parental P peptide, ^65^HGFLPLK^71^, detected at *m/z* = 406.3 in the charge state of +2. (G) Tandem mass spectrum assigned to the predicted *b*- and *y*-ions generated from collision-induced fragmentation of the P* peptide, ^65^HGFLP**L^U^K^A^** ^71^, detected at *m/z* = 662.2 in the charge state of +2 with the L70 and K71 residues bound to uracil (U) and adenine (A), respectively. (H) Tandem mass spectrum assigned to the predicted *b*- and *y*-ions generated from collision-induced fragmentation of a parental M peptide, ^427^LIEEEALK^433^, detected at *m/z* = 473.0 in the charge state of +2. (I) Tandem mass spectrum assigned to the predicted *b*- and *y*-ions generated from collision-induced fragmentation of the M* peptide, ^427^LIEEEAL**K** ^C433^, detected at *m/z* = 594.5 in the charge state of +2 with the K433 residue bound to cytosine (C). **Figure S3. Detection of misfolding of N-Rne mutants.** Purified proteins of N-Rne, N-Rne-Y25A, and N-Rne-Q36R were used to measure the CD spectrum. **Table S1.** **Results of nLC-tandem mass spectrometry analyses of wild-type N-Rne, Y25A and Q36R mutant samples crosslinked with p-BR13.** *Note.* Variable modifications at the positions with the lowercase one-letter amino acid codes: CAM, carbamidomethylation (C); O, oxidation (M, W); and UV crosslinked residues with nucleosides (A, U or C) in parentheses.
(DOCX)
