# Supplementary material for: Modulation of RNase E Activity by Alternative RNA Binding Sites
Source: PLoS One. 2014 Mar 5;9(3):e90610. doi: 10.1371/journal.pone.0090610 (PMC3944109; doi:10.1371/journal.pone.0090610)
Supplement: File S1 — Supporting Figures S1–S3 and Table S1. Figure S1. Base peak chromatograms of UV-crosslinked monomer samples of wild-type and mutant N-Rne proteins. Analyzed by nLC-LTQ Velos mass spectrometry between 60 to 90 min over a mass range of 300 to 2,000. After UV-crosslinking, the monomer bands were excised from SDS PAGE gels as described in the main text. Figure S2. Tandem mass spectra and assignment of b - and y -ions of peptides bound to p-BR13. The parent peptides, 26DLDIESPGHEQK37, 65HGFLPLK71, and 427LIEEEALK433, are denoted as R, P, and M, respectively, and show an asterisk to the right of the letter to indicate the chemical binding of nucleosides by UV-crosslinking between protein and p-BR13 in the extracted ion chromatograms (panels a, c and e) and the corrected PSM levels (panels b, d and f). A unique stripped peptide,16VALVDGQR23, used as the internal standard (IS) for calculation of relative intensity or corrected PSM level was included as below. (A) Tandem mass spectrum assigned to the predicted b- and y-ions generated from collision-induced fragmentation of the stripped IS peptide, 16VALVDGQR23, at m/z = 429.5 in a charge state of +2. (B) Tandem mass spectrum assigned to the predicted b- and y-ions generated from collision-induced fragmentation of a parental RWT peptide of the wild-type N-Rne, 26DLDIESPGHEQK37, detected at m/z = 684.7 in the charge state of +2. (C) Tandem mass spectrum assigned to the predicted b- and y-ions generated from collision-induced fragmentation of the RWT* peptide, 26 D A L CDIESPGHEQK37, detected at m/z = 627.0 in the charge state of +3 with the D26 and L27 residues bound to adenine (A) and cytosine (C), respectively. (D) Tandem mass spectrum assigned to the predicted b- and y-ions generated from collision-induced fragmentation of a parental RY25A peptide of the Y25A mutant, 24LADLDIESPGHEQK37, detected at m/z = 776.6 in the charge state of +2. (E) Tandem mass spectrum assigned to the predicted b- and y-ions generated from collisio [file pone.0090610.s001.docx]

**Table S1.** Results of nLC-tandem mass spectrometry analyses of wild-type N-Rne, Y25A and Q36R mutant samples crosslinked with p-BR13.

| Protein | # Unique Peptides | PSMs | %Coverage |  |  |  |  |  |  | |  | |  |
| --- | --- | --- | --- | --- | --- | --- | --- | --- | --- | --- | --- | --- | --- |
| N-Rne | 44 | 1129 | 65.94 |  |  |  |  |  |  | |  | |  |
|  | Sequence | PSMs | Modifications | XCorr | SpScore | z | MH+ [Da] | ΔM [ppm] | m/z [Da] | | RT [min] | |  |
|  | LYDLDIESPGHEQK | 14 |  | 3.92 | 1407.36 | 2 | 1644.30 | -291.83 | 822.65 | | 78.44 | |  |
|  | EALASLELPEGmGLIVR | 23 | M12(O) | 3.88 | 648.92 | 2 | 1815.44 | 165.37 | 908.23 | | 87.35 | |  |
|  | EGQEVIVQIDKEER | 34 |  | 3.61 | 1194.07 | 2 | 1672.19 | -382.98 | 836.60 | | 75.86 | |  |
|  | ISLAGSYLVLmPNNPR | 7 | M11(O) | 3.32 | 977.05 | 2 | 1762.27 | 106.45 | 881.64 | | 85.75 | |  |
|  | mLINATQQEELR | 59 | M1(O) | 3.27 | 1177.55 | 2 | 1462.17 | -339.62 | 731.59 | | 75.60 | |  |
|  | MLINATQQEELR | 19 |  | 3.14 | 1538.63 | 2 | 1446.11 | -389.64 | 723.56 | | 78.36 | |  |
|  | QHIAALGRPDFSSK | 26 |  | 3.10 | 451.19 | 2 | 1527.16 | -372.19 | 764.08 | | 73.08 | |  |
|  | LIHQESNVIVR | 67 |  | 3.03 | 1135.84 | 3 | 1308.56 | 27.98 | 436.86 | | 70.64 | |  |
|  | QDIGEILIDNPK | 51 |  | 2.99 | 1167.03 | 2 | 1354.85 | -505.65 | 677.93 | | 82.16 | |  |
|  | EALASLELPEGMGLIVR | 4 |  | 2.83 | 538.39 | 2 | 1798.44 | -393.51 | 899.72 | | 92.42 | |  |
|  | DNESLSLSILR | 24 |  | 2.72 | 956.21 | 2 | 1247.30 | -77.30 | 624.15 | | 85.44 | |  |
|  | DLDIESPGHEQK | 8 |  | 2.70 | 1135.39 | 2 | 1367.36 | -790.00 | 684.19 | | 71.07 | |  |
|  | LSPSLGESSHHVcPR | 27 | C13(CAM) | 2.66 | 614.30 | 2 | 1663.09 | -458.92 | 832.05 | | 69.66 | |  |
|  | RSAVNAIETR | 28 |  | 2.55 | 597.71 | 2 | 1116.93 | -291.60 | 558.97 | | 64.56 | |  |
|  | TGEIPLFSHY | 9 |  | 2.54 | 592.58 | 2 | 1163.97 | -288.68 | 582.49 | | 84.31 | |  |
|  | GNKGAALTTF | 7 |  | 2.43 | 858.44 | 2 | 979.90 | -213.17 | 490.45 | | 73.16 | |  |
|  | ENTQEVHAIVPVPIASY | 6 |  | 2.39 | 479.80 | 2 | 1868.42 | 171.11 | 934.71 | | 86.59 | |  |
|  | QIESQIESAFQR | 34 |  | 2.39 | 486.31 | 2 | 1437.02 | 315.90 | 719.01 | | 77.70 | |  |
|  | SAVNAIETR | 61 |  | 2.33 | 1298.46 | 2 | 960.26 | -838.37 | 480.63 | | 85.32 | |  |
|  | DLGGLIVIDFIDmTPVR | 2 | M13(O) | 2.29 | 794.48 | 2 | 1890.62 | -328.43 | 945.81 | | 96.73 | |  |
|  | NTNLEAADEIAR | 14 |  | 2.23 | 708.40 | 2 | 1317.83 | 328.83 | 659.42 | | 76.19 | |  |
|  | cVIVPNDQmETPHY | 1 | C1(CAM); M9(O) | 2.20 | 301.95 | 2 | 1720.01 | 41.15 | 860.51 | | 77.00 | |  |
|  | FGLLEmSR | 30 | M6(O) | 2.18 | 959.65 | 2 | 968.79 | -375.91 | 484.90 | | 77.79 | |  |
|  | HGFLPLK | 54 |  | 2.14 | 919.60 | 2 | 811.75 | -313.90 | 406.38 | | 106.13 | |  |
|  | FGLLEMSR | 12 |  | 2.10 | 1149.47 | 2 | 952.21 | -994.33 | 476.61 | | 82.03 | |  |
|  | EGQEVIVQIDK | 4 |  | 2.09 | 773.11 | 2 | 1257.96 | -362.75 | 629.48 | | 76.25 | |  |
|  | LVLmPNNPR | 25 | M4(O) | 2.09 | 742.17 | 2 | 1070.02 | -263.65 | 535.51 | | 72.08 | |  |
|  | LIEEEALK | 24 |  | 2.06 | 439.80 | 2 | 944.28 | -875.10 | 472.64 | | 72.76 | |  |
|  | SAVNAIETRQDGVR | 8 |  | 2.06 | 556.63 | 3 | 1516.29 | -243.17 | 506.10 | | 71.32 | |  |
|  | SAHGRPNIK | 194 |  | 2.06 | 321.09 | 2 | 979.23 | -906.35 | 490.12 | | 25.63 | |  |
|  | IEPSLEAAFVDY | 21 |  | 2.05 | 697.80 | 2 | 1354.09 | -300.56 | 677.55 | | 89.18 | |  |
|  | LIEEEALk | 28 | K8(C) | 1.94 | 965.80 | 2 | 1187.87 | -375.64 | 594.44 | | 73.73 | |  |
|  | IEGDDRTELK | 35 |  | 1.93 | 743.53 | 2 | 1175.93 | -289.38 | 588.47 | | 63.63 | |  |
|  | IQISHISR | 59 |  | 1.92 | 633.53 | 2 | 953.78 | -352.59 | 477.39 | | 68.21 | |  |
|  | LRQDIGEILIDNPK | 2 |  | 1.92 | 263.34 | 2 | 1624.51 | -229.73 | 812.76 | | 80.97 | |  |
|  | KAAESRPAPF | 9 |  | 1.85 | 590.51 | 2 | 1073.82 | -375.86 | 537.41 | | 66.39 | |  |
|  | dlDIESPGHEQK | 3 | D1(A); L2(C) | 1.84 | 506.38 | 3 | 1878.91 | 2.29 | 626.97 | | 68.57 | |  |
|  | SAEALQw | 2 | W7(O) | 1.75 | 665.87 | 2 | 819.95 | -1132.69 | 410.48 | | 67.68 | |  |
|  | VALVDGQR | 69 |  | 1.69 | 926.25 | 2 | 857.90 | -99.53 | 429.45 | | 66.38 | |  |
|  | DLDIESPGHEQKK | 4 |  | 1.67 | 489.09 | 2 | 1496.05 | -378.33 | 748.53 | | 68.15 | |  |
|  | LVLMPNNPR | 9 |  | 1.64 | 471.47 | 2 | 1053.90 | -381.88 | 527.45 | | 76.52 | |  |
|  | GAALTTFISLAGSY | 4 |  | 1.63 | 314.83 | 2 | 1371.96 | -435.54 | 686.49 | | 92.49 | |  |
|  | HGFLpLk | 6 | L6(U); K7(A) | 1.63 | 259.97 | 2 | 1323.13 | -240.64 | 662.07 | | 82.59 | |  |
|  | QHIAALGRPDF | 2 |  | 1.53 | 220.27 | 2 | 1224.13 | -1031.78 | | 612.57 | | 76.49 | |
| Protein | # Unique Peptides | PSMs | %Coverage |  |  |  |  |  |  | |  | |  |
| Y25A | 47 | 1195 | 67.72 |  |  |  |  |  |  | |  | |  |
|  | Sequence | PSMs | Modifications | XCorr | SpScore | z | MH+ [Da] | ΔM [ppm] | m/z [Da] | | RT [min] | |  |
|  | EALASLELPEGMGLIVR | 5 |  | 3.84 | 741.94 | 2 | 1798.31 | -465.09 | 899.66 | | 92.73 | |  |
|  | EGQEVIVQIDKEER | 53 |  | 3.63 | 824.09 | 2 | 1672.20 | -378.31 | 836.60 | | 76.21 | |  |
|  | ISLAGSYLVLmPNNPR | 8 | M11(O) | 3.42 | 946.42 | 2 | 1760.96 | -642.13 | 880.98 | | 85.47 | |  |
|  | MLINATQQEELR | 22 |  | 3.39 | 1503.50 | 2 | 1446.25 | -290.85 | 723.63 | | 77.24 | |  |
|  | LADLDIESPGHEQKK | 18 |  | 3.38 | 588.27 | 2 | 1680.26 | -357.32 | 840.63 | | 72.62 | |  |
|  | EALASLELPEGmGLIVR | 17 | M12(O) | 3.20 | 491.16 | 2 | 1814.46 | -379.35 | 907.73 | | 104.55 | |  |
|  | LRQDIGEILIDNPK | 1 |  | 3.16 | 958.90 | 2 | 1624.29 | -360.38 | 812.65 | | 80.52 | |  |
|  | LADLDIESPGHEQK | 30 |  | 3.11 | 1203.13 | 2 | 1552.14 | -350.05 | 776.57 | | 74.99 | |  |
|  | QHIAALGRPDFSSK | 30 |  | 3.06 | 321.24 | 2 | 1527.10 | -409.45 | 764.05 | | 72.60 | |  |
|  | DNESLSLSILR | 32 |  | 2.98 | 1364.58 | 2 | 1246.44 | -765.65 | 623.72 | | 82.38 | |  |
|  | QDIGEILIDNPK | 31 |  | 2.88 | 1043.41 | 2 | 1355.21 | -239.52 | 678.11 | | 81.66 | |  |
|  | mLINATQQEELR | 60 | M1(O) | 2.88 | 1546.56 | 2 | 1462.12 | -378.87 | 731.56 | | 74.96 | |  |
|  | QIESQIESAFQR | 41 |  | 2.78 | 789.74 | 2 | 1437.26 | 485.71 | 719.14 | | 81.31 | |  |
|  | SAVNAIETR | 66 |  | 2.62 | 1069.00 | 2 | 960.93 | -141.45 | 480.97 | | 68.69 | |  |
|  | HGFLPLK | 75 |  | 2.59 | 997.04 | 2 | 811.91 | -117.11 | 406.46 | | 74.84 | |  |
|  | NTNLEAADEIAR | 8 |  | 2.55 | 1203.19 | 2 | 1318.01 | 466.78 | 659.51 | | 74.06 | |  |
|  | TGEIPLFSHY | 7 |  | 2.42 | 505.43 | 2 | 1164.08 | -193.96 | 582.54 | | 84.03 | |  |
|  | LSPSLGESSHHVcPR | 18 | C13(CAM) | 2.39 | 459.20 | 2 | 1663.22 | -383.44 | 832.11 | | 67.62 | |  |
|  | DLGGLIVIDFIDmTPVR | 1 | M13(O) | 2.34 | 714.67 | 2 | 1890.72 | -276.50 | 945.86 | | 96.66 | |  |
|  | GEETPTLSHHHHHH | 10 |  | 2.33 | 324.38 | 3 | 1656.48 | -143.22 | 552.83 | | 55.06 | |  |
|  | KGEETPTLSHHHHHH | 15 |  | 2.32 | 516.22 | 3 | 1784.01 | -494.43 | 595.34 | | 54.45 | |  |
|  | ARIQISHISR | 2 |  | 2.27 | 480.66 | 3 | 1181.34 | -35.71 | 394.45 | | 67.32 | |  |
|  | IEGDDRTELK | 32 |  | 2.22 | 1076.34 | 2 | 1175.88 | -330.71 | 588.44 | | 63.35 | |  |
|  | LIHQESNVIVR | 84 |  | 2.21 | 459.29 | 2 | 1307.81 | -548.88 | 654.41 | | 69.63 | |  |
|  | RSAVNAIETR | 34 |  | 2.09 | 719.67 | 2 | 1116.88 | -329.64 | 558.95 | | 63.54 | |  |
|  | GNKGAALTTF | 5 |  | 2.08 | 869.54 | 2 | 979.84 | -274.73 | 490.42 | | 72.87 | |  |
|  | LIEEEALK | 16 |  | 2.08 | 377.63 | 2 | 944.99 | -117.79 | 473.00 | | 72.39 | |  |
|  | SAHGRPNIK | 148 |  | 2.05 | 364.07 | 2 | 979.19 | -943.66 | 490.10 | | 17.00 | |  |
|  | QIESQIESAF | 11 |  | 2.03 | 626.29 | 2 | 1152.07 | -153.50 | 576.54 | | 82.54 | |  |
|  | ENTQEVHAIVPVPIASY | 7 |  | 2.03 | 557.31 | 2 | 1867.35 | -398.82 | 934.18 | | 85.81 | |  |
|  | SAVNAIETRQDGVR | 8 |  | 2.01 | 843.90 | 3 | 1516.98 | 215.01 | 506.33 | | 70.73 | |  |
|  | IQISHISR | 43 |  | 2.01 | 708.98 | 2 | 953.88 | -252.55 | 477.44 | | 68.12 | |  |
|  | FGLLEmSR | 25 | M6(O) | 2.00 | 921.24 | 2 | 968.82 | -340.37 | 484.91 | | 77.36 | |  |
|  | IEPSLEAAFVDY | 17 |  | 1.98 | 763.57 | 2 | 1354.13 | -276.31 | 677.57 | | 89.07 | |  |
|  | GAALTTFISLAGSY | 1 |  | 1.96 | 542.08 | 2 | 1371.96 | -440.26 | 686.48 | | 92.62 | |  |
|  | EGQEVIVQIDK | 5 |  | 1.88 | 839.08 | 2 | 1258.09 | -257.73 | 629.55 | | 76.04 | |  |
|  | LIEEEALk | 24 | K8(C) | 1.88 | 806.24 | 2 | 1188.06 | -221.05 | 594.53 | | 73.68 | |  |
|  | IDmTPVR | 7 | M3(O) | 1.85 | 630.83 | 2 | 847.82 | -222.86 | 424.42 | | 62.59 | |  |
|  | KAAESRPAPF | 13 |  | 1.85 | 757.29 | 2 | 1073.76 | -433.41 | 537.38 | | 65.61 | |  |
|  | FGLLEMSR | 17 |  | 1.75 | 1021.28 | 2 | 952.78 | -387.98 | 476.89 | | 81.76 | |  |
|  | LVLMPNNPR | 5 |  | 1.73 | 588.55 | 2 | 1053.94 | -340.86 | 527.48 | | 75.82 | |  |
|  | QHIAALGRPDF | 3 |  | 1.72 | 239.17 | 2 | 1224.22 | -958.22 | 612.61 | | 76.07 | |  |
|  | VLELAR | 21 |  | 1.72 | 365.69 | 2 | 700.70 | -219.38 | 350.86 | | 69.69 | |  |
|  | VALVDGQR | 59 |  | 1.68 | 807.29 | 2 | 857.82 | -189.98 | 429.42 | | 66.58 | |  |
|  | HGFLPlk | 12 | L6(U); K7(A) | 1.63 | 501.70 | 2 | 1323.25 | -147.07 | 662.13 | | 82.59 | |  |
|  | EYFPANY | 19 |  | 1.56 | 587.50 | 2 | 903.90 | -77.56 | 452.45 | | 78.88 | |  |
|  | LVLmPNNPR | 29 | M4(O) | 1.50 | 499.84 | 2 | 1069.96 | -322.65 | 535.48 | | 71.40 | |  |
| Protein | # Unique Peptides | PSMs | %Coverage |  |  |  |  |  |  | |  | |  |
| Q36R | 49 | 1646 | 71.49 |  |  |  |  |  |  | |  | |  |
|  | Sequence | PSMs | Modifications | XCorr | SpScore | z | MH+ [Da] | ΔM [ppm] | m/z [Da] | | RT [min] | |  |
|  | EALASLELPEGmGLIVR | 58 | M12(O) | 3.79 | 620.76 | 2 | 1814.39 | -414.08 | 907.70 | | 87.35 | |  |
|  | LYDLDIESPGHER | 89 |  | 3.37 | 1411.91 | 2 | 1544.11 | -360.27 | 772.56 | | 78.79 | |  |
|  | LRQDIGEILIDNPK | 2 |  | 3.30 | 1002.64 | 2 | 1625.10 | 134.66 | 813.05 | | 80.53 | |  |
|  | EALASLELPEGMGLIVR | 16 |  | 3.13 | 528.02 | 2 | 1798.44 | -389.98 | 899.73 | | 92.41 | |  |
|  | DNESLSLSILR | 45 |  | 3.07 | 940.80 | 2 | 1247.99 | 480.08 | 624.50 | | 85.14 | |  |
|  | QHIAALGRPDFSSK | 37 |  | 3.04 | 324.72 | 2 | 1527.09 | -413.93 | 764.05 | | 72.79 | |  |
|  | mLINATQQEELR | 89 | M1(O) | 2.99 | 1067.64 | 2 | 1462.02 | -443.43 | 731.51 | | 71.50 | |  |
|  | MLINATQQEELR | 29 |  | 2.90 | 1077.32 | 2 | 1446.08 | -410.25 | 723.54 | | 77.11 | |  |
|  | QDIGEILIDNPK | 51 |  | 2.86 | 1026.51 | 2 | 1355.17 | -267.63 | 678.09 | | 103.60 | |  |
|  | ISLAGSYLVLmPNNPR | 8 | M11(O) | 2.84 | 728.20 | 2 | 1761.29 | -453.08 | 881.15 | | 85.60 | |  |
|  | QIESQIESAFQR | 56 |  | 2.84 | 1396.09 | 2 | 1437.04 | 327.20 | 719.02 | | 81.47 | |  |
|  | RSAVNAIETR | 35 |  | 2.80 | 701.35 | 2 | 1116.88 | -331.50 | 558.94 | | 63.74 | |  |
|  | RmLINATQQEELR | 1 | M2(O) | 2.74 | 1035.63 | 3 | 1618.64 | -133.16 | 540.22 | | 72.28 | |  |
|  | KGEETPTLSHHHHHH | 15 |  | 2.73 | 843.50 | 3 | 1784.81 | -47.74 | 595.61 | | 54.87 | |  |
|  | NTNLEAADEIAR | 11 |  | 2.71 | 1670.84 | 2 | 1316.90 | -380.33 | 658.95 | | 74.52 | |  |
|  | LIHQESNVIVR | 99 |  | 2.68 | 426.99 | 2 | 1308.01 | -391.63 | 654.51 | | 69.79 | |  |
|  | LSPSLGESSHHVcPR | 35 | C13(CAM) | 2.66 | 572.49 | 2 | 1663.06 | -476.84 | 832.03 | | 68.98 | |  |
|  | DLGGLIVIDFIDmTPVR | 4 | M13(O) | 2.61 | 1089.54 | 2 | 1890.52 | -381.78 | 945.76 | | 96.73 | |  |
|  | EGQEVIVQIDKEER | 81 |  | 2.43 | 526.24 | 2 | 1672.37 | -279.58 | 836.69 | | 77.34 | |  |
|  | SAVNAIETRQDGVR | 13 |  | 2.39 | 576.73 | 3 | 1515.85 | -532.54 | 505.95 | | 70.43 | |  |
|  | SHYQIESQIESAF | 1 |  | 2.29 | 694.13 | 2 | 1539.37 | -178.56 | 770.19 | | 83.29 | |  |
|  | TGEIPLFSHY | 11 |  | 2.21 | 490.02 | 2 | 1164.09 | -187.46 | 582.55 | | 84.07 | |  |
|  | DLDIESPGHER | 13 |  | 2.20 | 717.47 | 2 | 1268.02 | -239.06 | 634.52 | | 71.36 | |  |
|  | HGFLPLK | 64 |  | 2.19 | 862.05 | 2 | 811.66 | -421.99 | 406.33 | | 76.38 | |  |
|  | ENTQEVHAIVPVPIASY | 11 |  | 2.17 | 395.35 | 2 | 1867.37 | -389.02 | 934.19 | | 85.58 | |  |
|  | GNKGAALTTF | 12 |  | 2.16 | 881.80 | 2 | 979.85 | -266.25 | 490.43 | | 72.87 | |  |
|  | LIEEEALk | 26 | K8(C) | 2.15 | 1107.98 | 2 | 1188.02 | -249.62 | 594.51 | | 73.24 | |  |
|  | cVIVPNDQmETPHY | 1 | C1(CAM); M9(O) | 2.14 | 414.10 | 2 | 1719.96 | 14.74 | 860.49 | | 76.70 | |  |
|  | EGQEVIVQIDK | 6 |  | 2.07 | 1085.54 | 2 | 1258.14 | -217.35 | 629.57 | | 76.14 | |  |
|  | IEGDDRTELK | 44 |  | 2.03 | 853.96 | 2 | 1176.01 | -222.30 | 588.51 | | 62.81 | |  |
|  | GAALTTFISLAGSY | 10 |  | 2.01 | 532.53 | 2 | 1372.21 | -259.60 | 686.61 | | 92.62 | |  |
|  | IEPSLEAAFVDY | 25 |  | 2.00 | 1052.20 | 2 | 1354.23 | -201.29 | 677.62 | | 89.08 | |  |
|  | FGLLEmSR | 34 | M6(O) | 1.95 | 989.23 | 2 | 968.83 | -332.43 | 484.92 | | 77.04 | |  |
|  | SAVNAIETR | 81 |  | 1.95 | 942.41 | 2 | 960.61 | -472.98 | 480.81 | | 67.69 | |  |
|  | LVLMPNNPR | 11 |  | 1.89 | 516.24 | 2 | 1053.98 | -304.95 | 527.49 | | 75.94 | |  |
|  | KAAESRPAPF | 10 |  | 1.88 | 545.59 | 2 | 1073.94 | -265.11 | 537.47 | | 65.58 | |  |
|  | SAHGRPNIKDVLR | 8 |  | 1.88 | 458.31 | 3 | 1462.93 | -513.61 | 488.32 | | 67.27 | |  |
|  | VALVDGQR | 73 |  | 1.81 | 889.48 | 2 | 857.81 | -210.26 | 429.41 | | 66.66 | |  |
|  | SAHGRPNIK | 222 |  | 1.79 | 259.65 | 2 | 979.81 | -306.87 | 490.41 | | 17.83 | |  |
|  | SAEALQw | 5 | W7(O) | 1.79 | 723.30 | 2 | 819.68 | -1457.41 | 410.35 | | 67.12 | |  |
|  | LPLKEIAR | 10 |  | 1.77 | 332.01 | 2 | 939.96 | -231.39 | 470.48 | | 71.32 | |  |
|  | EYFPANY | 21 |  | 1.75 | 648.10 | 2 | 903.74 | -257.28 | 452.37 | | 79.05 | |  |
|  | GEETPTLSHHHHHH | 9 |  | 1.75 | 467.08 | 3 | 1656.29 | -260.31 | 552.77 | | 55.58 | |  |
|  | FGLLEMSR | 24 |  | 1.71 | 967.43 | 2 | 952.30 | -896.05 | 476.65 | | 82.06 | |  |
|  | VLELAR | 28 |  | 1.66 | 306.91 | 2 | 700.82 | -50.38 | 350.91 | | 69.43 | |  |
|  | LVLmPNNPR | 30 | M4(O) | 1.64 | 433.90 | 2 | 1069.98 | -305.65 | 535.49 | | 71.78 | |  |
|  | HGFLPlk | 26 | L6(U); K7(A) | 1.63 | 501.70 | 2 | 1323.25 | -147.07 | 662.13 | | 82.59 | |  |
|  | RIEGDDR | 31 |  | 1.60 | 414.51 | 2 | 860.92 | 24.79 | 430.97 | | 5.82 | |  |
|  | LIEEEALK | 25 |  | 1.55 | 597.69 | 1 | 944.51 | -627.24 | 944.51 | | 72.35 | |  |

*Note.* Variable modifications at the positions with the lowercase one-letter amino acid codes: CAM, carbamidomethylation (C); O, oxidation (M, W); and UV crosslinked residues with nucleosides (A, U or C) in parentheses.

**Figure S1. Base peak chromatograms of UV-crosslinked monomer samples of wild type and mutant N-Rne proteins.** Analysis was performed using an nLC-LTQ Velos mass spectrometer operated between 60 to 90 min over a full-mass range of *m/z* 300 and 2,000. After UV-crosslinking, the monomer bands were excised from SDS PAGE gels and treated as described in the main text.

**Figure S2(A-I). Tandem mass spectra and assignment of *b*- and *y*-ions of peptides bound and unbound to p-BR13.** The precursor mass peaks of the three parent peptides, ^26^DLDIESPGHEQK^37^, ^65^HGFLPLK^71^, and ^427^LIEEEALK^433^ denoted with the respective symbols of R, P, and M, are found in the extracted ion chromatograms (panels a, c and e) and the corrected PSM levels (panels b, d and f) of Figure 4 and their crosslinks with p-BR13 are indicated by asterisks to the rights of the symbols. A unique stripped peptide,^16^VALVDGQR^23^, was used as the internal standard (**IS**) for the calculation of relative intensity or corrected PSM level.

**(A)** Tandem mass spectrum assigned to the predicted *b*- and *y*-ions generated from collision-induced fragmentation of the stripped **IS** peptide, ^16^VALVDGQR^23^, at *m/z* = 429.5 in a charge state of +2.

**(B)** Tandem mass spectrum assigned to the predicted *b*- and *y*-ions generated from collision-induced fragmentation of a parental R_WT_ peptide of the wild-type N-Rne, ^26^DLDIESPGHEQK^37^, detected at *m/z* = 684.7 in the charge state of +2.

**(C)** Tandem mass spectrum assigned to the predicted *b*- and *y*-ions generated from collision-induced fragmentation of the R_WT_* peptide, ^26^**D**^A^**L**^C^DIESPGHEQK^37^, detected at *m/z* = 627.0 in the charge state of +3 with the D26 and L27 residues bound to adenine (A) and cytosine (C), respectively.

**(D)** Tandem mass spectrum assigned to the predicted *b*- and *y*-ions generated from collision-induced fragmentation of a parental R_Y25A_ peptide of the Y25A mutant, ^24^LADLDIESPGHEQK^37^, detected at *m/z* = 776.6 in the charge state of +2.

**(E)** Tandem mass spectrum assigned to the predicted *b*- and *y*-ions generated from collision-induced fragmentation of a parental R_Q36R_ peptide of the Q36R mutant, ^26^DLDIESPGHER^36^, detected at *m/z* = 634.5 in the charge state of +2.

**(F)** Tandem mass spectrum assigned to the predicted *b*- and *y*-ions generated from collision-induced fragmentation of a parental P peptide, ^65^HGFLPLK^71^, detected at *m/z* = 406.3 in the charge state of +2.

**(G)** Tandem mass spectrum assigned to the predicted *b*- and *y*-ions generated from collision-induced fragmentation of the P* peptide, ^65^HGFLP**L^U^K^A^**^71^, detected at *m/z* = 662.2 in the charge state of +2 with the L70 and K71 residues bound to uracil (U) and adenine (A), respectively.

**(H)** Tandem mass spectrum assigned to the predicted *b*- and *y*-ions generated from collision-induced fragmentation of a parental M peptide, ^427^LIEEEALK^433^, detected at *m/z* = 473.0 in the charge state of +2.

**(I)** Tandem mass spectrum assigned to the predicted *b*- and *y*-ions generated from collision-induced fragmentation of the M* peptide, ^427^LIEEEAL**K**^C433^, detected at *m/z* = 594.5 in the charge state of +2 with the K433 residue bound to cytosine (C).

**Figure S3. Detection of misfolding of N-Rne mutants.** Purified proteins of N-Rne, N-Rne-Y25A, and N-Rne-Q36R were used to measure the CD spectrum.
